# Community-acquired *Staphylococcus aureus* bacteriuria: a warning microbiological marker for infective endocarditis?

**DOI:** 10.1186/s12879-019-4106-0

**Published:** 2019-06-07

**Authors:** Thomas Lafon, Ana Catalina Hernandez Padilla, Arthur Baisse, Lucie Lavaud, Marine Goudelin, Olivier Barraud, Thomas Daix, Bruno Francois, Philippe Vignon

**Affiliations:** 1 Inserm CIC 1435, Centre hospitalier universitaire Dupuytren, F-87042 Limoges, France; 2 Service d’Accueil des Urgences, Centre hospitalier universitaire Dupuytren, F-87042 Limoges, France; 3 Service de Réanimation Polyvalente, Centre hospitalier universitaire Dupuytren, 2 Avenue Martin Luther King, 87042 Limoges cedex, France; 4 Laboratoire de Bactériologie – Virologie – Hygiène, Centre hospitalier universitaire Dupuytren, F-87042 Limoges, France; 5 Inserm UMR 1092, Faculté de Médecine, Université Limoges, F-87042 Limoges, France

**Keywords:** Bacteriuria, *Staphylococcus aureus*, Bacterial endocarditis, Emergency department, Bacteremia

## Abstract

**Background:**

Urinary tract infection (UTI) is frequently diagnosed in the Emergency Department (ED). *Staphylococcus aureus* (SA) is an uncommon isolate in urine cultures (0.5–6% of positive urine cultures), except in patients with risk factors for urinary tract colonization. In the absence of risk factors, community-acquired SA bacteriuria may be related to deep-seated SA infection including infective endocarditis. We hypothesized that SA bacteriuria could be a warning microbiological marker of unsuspected infective endocarditis in the ED.

**Methods:**

This is a retrospective chart review of consecutive adult patients between December 2005 and February 2018. All patients admitted in the ED with both SA bacteriuria (10^4^ CFU/ml SA isolated from a single urine sample) and SA bacteremia, without risk factors for UT colonization (i.e., < 1 month UT surgery, UT catheterization) were analyzed. Diagnosis of infective endocarditis was based on the Duke criteria.

**Results:**

During the study period, 27 patients (18 men; median age: 61 [IQR: 52–73] years) were diagnosed with community-acquired SA bacteriuria and had subsequently documented bacteremia and SA infective endocarditis. Only 5 patients (18%) had symptoms related to UT infection. Median delay between ED admission and SA bacteriuria identification was significantly shorter than that between ED admission and the diagnosis of infective endocarditis (1.4 ± 0.8 vs. 4.3 ± 4.2 days: *p* = 0.01). Mitral and aortic valves were most frequently involved by infective endocarditis (93%). Mortality on day 60 reached 56%.

**Conclusions:**

This study suggests that community-acquired SA bacteriuria should warn the emergency physician about a potentially associated left-sided infective endocarditis in ED patients without risk factors for UT colonization.

## Background

*Staphylococcus aureus* (*SA*) is an unusual cause of urinary tract infection (UTI) which prevalence ranges between 0.15 and 4.3% [1]. *SA* bacteriuria has been described predominantly in patients with predisposing conditions for ascending *SA* colonization (e.g., history of urinary obstruction, urinary catheter, recent urological surgical procedures, malignancy and recent hospitalization) [1–3]. Nevertheless, it is commonly interpreted as a genitourinary infection [2–4]. In up to 34% of cases, *SA* bacteriuria is associated with *SA* bacteremia [1, 2, 5, 6]. These patients have frequently a complicated course with higher hospital mortality [3, 5–7].

In the absence of risk factors for *SA* colonization, *SA* bacteriuria may be related to deep-seated *SA* infection, and specifically to infective kidney embolisms associated with an underlying infective endocarditis [3, 6]. Accordingly, this observational study aimed at describing the clinical presentation of patients admitted to the Emergency Department (ED) with *SA* bacteriuria who were subsequently diagnosed with *SA* infective endocarditis.

## Methods

This case series is based on a retrospective chart review of consecutive adult patients admitted initially to the ED of our institution between December 2005 and February 2018, with *SA* bacteriuria identified in the ED but no risk factors for urinary tract colonization, and who were subsequently diagnosed with definitive infective endocarditis. Patients were studied when (i) admitted to the ED, with (ii) initial *SA* bacteriuria on the first urine sample, (iii) subsequent identification of *SA* bacteremia, and (iv) definite diagnosis of infective endocarditis confirmed at hospital discharge. According to the French law, approval from an ethics committee, as well as consent to participate were not required since this study was retrospective and performed on existing data without direct intervention on human subjects.

Bacteriuria was defined as a positive culture > 10^4^ CFU/ml *SA* isolated from a single urine sample. Risk factors for urinary tract colonization included a history of urinary obstruction, urinary catheter, recent urological surgical procedures, malignancy and recent hospitalization. Concomitant bacteremia was defined as at least one blood culture positive for *SA* within 72 h following urine culture [2]. Diagnosis of definite infective endocarditis was based on the presence of typical echocardiographic findings (in conjunction of *SA* bacteremia) and modified Duke’s criteria were secondarily used to precisely determine major and minor criteria [8]. All patients underwent a transthoracic or transesophageal echocardiography. Demographic data, presence of a urinary catheter or inserted device, main reason for ED admission, UTI symptoms [9], diagnosis at ED discharge, source of bacteremia, *SA* antibiotic susceptibility, time lag between ED admission and diagnosis of infective endocarditis, characteristics of infective endocarditis, and 2-month mortality were collected (from shared medical file).

Mann-Whitney test was used to compare the difference in delay between bacteriuria and diagnosis of infective endocarditis by echocardiography.

## Results

During the study period, 27 patients (18 men; median age: 61 [IQR: 52–73] years) were diagnosed in the ED with a community acquired *SA* bacteriuria and had subsequently documented bacteremia and *SA* definitive infective endocarditis. Median Charlson index was 2.0 [IQR: 1–4.5] and comorbidities included valve disease (30%), prosthetic valve surgery (18%), diabetes (18%), implantable catheter (18%), cirrhosis (11%) and cancer (7%). A single patient was an intravenous drug user (Table 1). Patients presented to the ED after a mean of 4 ± 3 days after the onset of symptoms, mostly with fever (59%) and confusion (48%). Only 5 patients (18%) had symptoms related to UTI (dysuria: *n* = 1; hematuria: *n* = 2; lumbar pain: *n* = 2). Five patients (18%) had a cardiac murmur, three having also a prosthetic valve. All patients were hospitalized. At ED discharge, 21 patients (78%) had a diagnosis of acute infection which site was identified in 14 patients, but only 5 of them were diagnosed out of hand with infective endocarditis (Table 1). *SA* strains were methicillin-susceptible (MSSA) in 25 patients (93%). The source of *SA* infection was documented in 59% of patients and was mainly of cutaneous or joint origin (Table 1).

**Table 1:** Characteristics of study population

Patient	Age-range (10 yr)	Gender	Valvulopathy/prosthetic valve	Diagnosis at ED discharge	Number of positive blood cultures (n)	Echocardiographic findings of infective endocarditis	Predisposing factors	Fever > 38 °C	Vascular phenomena	Immunologic phenomena	Infective endocarditis localization	Vegetation’s size (mm)^a^	Site of infection	Surgical indication	Mortality at day 60
1	20–30	F		Appendicitis	2	X			X		Mitral	UNK			
2	30–40	F		Endocarditis	11	X		X	X		Mitral	UNK	Skin	X	X
3	40–50	M		Sepsis	2	X		X	X		Aortic	UNK			
4	–	M		Epigastralgia	UNK	X			X		Mitral, Aortic	UNK	Skin		
5	–	M		Fever	UNK	X		X	X		Mitral, Aortic, Tricuspid	UNK	Skin	X	
6	50–60	M		Septic shock of UNK origin	6	X	X				Aortic	UNK			X
7	–	M	X	Septic shock of UNK origin	6	X	X	X			Tricuspid	UNK	Skin		X
8	–	M		Peritonitis	7	X			X		Mitral	23			X
9	–	M	X	Endocarditis	9	X	X	X	X		Mitral	UNK			X
10	–	F		Meningitis	10	X			X		Mitral	13	Skin		
11	–	M	X	Endocarditis	5	X	X	X	X		Mitral	15			X
12	–	F		Pyelonephritis	UNK	X		X	X		Mitral	7			
13	–	M		Septic shock of UNK origin	4	X			X	X	Mitral	12	Skin	X	
14	60–70	M	X	Sepsis	2	X	X	X	X	X	Mitral	20			
15	–	M	X	Chronic heart failure	4	X	X	X			Mitral	UNK	Skin		X
16	–	M	X	Endocarditis	2	X	X	X	X	X	Mitral	13	Skin	X	
17	–	M	X	Prostatitis	4	X	X	X	X		Mitral	18	Skin	X	X
18	70–80	F	X	Pneumonia	2	X	X	X			Mitral	UNK		X	X
19	–	M		Thoracic pain	3	X					Tricuspid	35	Skin		X
20	–	F	X	Endocarditis	2	X	X	X	X		Mitral	UNK	Joint		X
21	–	M		Dyspnea	2	X		X			Mitral	10	Skin		X
22	–	F		Diverticulitis	2	X		X	X	X	Mitral	23	Joint	X	
23	–	M		Pneumonia	2	X			X		Mitral	UNK	Skin		X
24	–	M	X	Asthenia	2	X	X				Mitral	15	Skin		
25	80–90	F		Low back pain	4	X			X	X	Mitral	UNK	Skin		X
26	–	F		Erysipelas	4	X		X	X	X	Aortic	13			
27	–	M		Dyspnea	4	X				X	Mitral, Aortic	8			X

^a^all patients had vegetations identified during echocardiographic assessment, but the size of the vegetation was not always measured

Abbreviations: *ED* Emergency Department, *UNK* Unknown

Mean delay between ED admission and *SA* bacteriuria identification was significantly shorter than that separating ED admission and the diagnosis of infective endocarditis (1.4 ± 3.1 vs. 4.3 ± 4.2 days: *p* = 0.0016). Moreover, *SA* bacteriuria from urine sample obtained in the ED was detected earlier than *SA* bacteremia with a mean difference of 1.9 days.

Mitral and aortic valves were most frequently involved by infective endocarditis (93%) (Table 1). Heart surgery was required in seven patients (4 men; median age: 65 years [IQR: 48–70]) after a median of 10 days (IQR: 7.5–23.5) from ED admission. Indications for surgery included uncontrolled infection despite adapted antibiotherapy (*n* = 3), massive valvular insufficiency (*n* = 5), or intractable pulmonary edema (*n* = 3). Mortality on day 60 reached 56%.

## Discussion

The clinical presentation of infective endocarditis is commonly non-specific and constitutes a syndromic diagnosis which should be suspected on the presence of compatible clinical signs associated with predisposing factors (e.g., valvulopathy, prosthetic valve, intravenous drug use), rather than on a single definitive test result [8]. In the present study, infective endocarditis was suspected mainly because of persistent bacteremia, but was also identified in the subset of shocked patients during initial bedside echocardiographic hemodynamic assessment. Median time from ED admission to diagnosis of infective endocarditis reached 3 [IQR 1–6.5] days, and was much shorter than that previously reported in the general population [10]. *SA* bacteriuria was identified even earlier (1.4 ± 0.8 days) after ED admission in all patients, and mostly in the absence of UTI symptoms [1, 4, 6]. This is presumably explained by the liberal use of urine culture in the ED [11]. Indeed, reasons for ED admission were not related to an infectious origin in more than half of the cases (59%) and diagnosis at ED discharge was non-infectious in 22% of patients. These results are in keeping with previous studies which reported initial misdiagnoses in 26 to 33% of patients with definite infective endocarditis [5].

Even in the absence of UTI, *SA* bacteremia is frequently (8 to 34%) accompanied by bacteriuria [1, 2, 4–6], and patients with both infectious events have an increased risk for complicated *SA* bacteremia [1, 7], and a two-fold increased risk for admission in intensive care unit (ICU) and hospital mortality [1, 6, 12]. In the present series, the mortality at 2 months after admission to ED was as high as 56%. This suggests that community-acquired *SA* bacteriuria detected in ED behaves as an early warning signal of potential poor outcome in patients with underlying infective endocarditis. In the present study, *SA* bacteriuria was related to complicated bacteremia secondary to bone/joint infections in 37% of patients, a similar proportion to that previously reported [4, 7]. Importantly, *SA* bacteriuria was identified significantly earlier than infective endocarditis in all our patients, with a mean difference of 2.9 days. This time difference could allow initiating antibiotic therapy earlier and may reduce complications of *SA* bacteremia since a delay of 48 h in antibiotics initiation is a risk factor for metastatic infection [5]. Considering the increased rates of ICU admission and mortality and the non-specific clinical presentation of infective endocarditis [8], *SA* bacteriuria could be helpful to accelerate both its identification and treatment, when used as a potential indicator of associated *SA* bacteremia with renal micro-abscesses [1, 6, 13]. In keeping with this hypothesis, 19 out of 27 patients (70%) exhibited vascular phenomena which are consistent with multiple septic systemic emboli, including the development of renal micro-abscesses with associated *SA* bacteriuria. Accordingly, MSSA, which is most frequently isolated in patients with metastatic *SA* bacteremia [2, 4], was predominantly identified and infective endocarditis involved left-sided valves in most of the herein reported cases.

The present study lacks power to ascertain the association between *SA* bacteriuria, *SA* bacteremia and infective endocarditis. In addition, its retrospective design precluded evaluating patients with isolated *SA* bacteriuria but no bacteremia to determine the specificity of this potential microbiological “marker”. Similarly, the sensitivity of *SA* bacteriuria could not be assessed since all patients with *SA* bacteremia failed to undergo urine culture at the time of blood culture sampling. Accordingly, these preliminary data need to be prospectively confirmed in a larger multicenter cohort of ED patients. However, data were collected and analyzed exhaustively by an independent adjudication committee.

## Conclusion

In closing, this study suggests that community-acquired *SA* bacteriuria should not be interpreted as an isolated UTI or colonization of urinary tract in the absence of risk factors, but should rather warn the front-line physician about a potential associated *SA* bacteremia secondary to a left-sided infective endocarditis. Whether this simple and easily accessible microbiological “marker” allows reducing the delay of both diagnosis and treatment of infective endocarditis in patients presenting to the ED with undifferentiated symptoms remains to be confirmed by prospective large-scale studies.

## Data Availability

The datasets analyzed during the current study are not publicly available since they contain confidential personal information that could help identify the patients but can be available from the corresponding author on reasonable request and after removal of all personal information.

## Abbreviations

ED: Emergency department
ICU: Intensive care unit
IQR: Interquartile range
MSSA: Methicillin-susceptible *Staphylococcus aureus*
SA: *Staphylococcus aureus*
UTI: Urinary tract infection
